# Bacterial Luciferases from *Vibrio harveyi* and *Photobacterium leiognathi* Demonstrate Different Conformational Stability as Detected by Time-Resolved Fluorescence Spectroscopy

**DOI:** 10.3390/ijms221910449

**Published:** 2021-09-28

**Authors:** Elena V. Nemtseva, Dmitry V. Gulnov, Marina A. Gerasimova, Lev A. Sukovatyi, Ludmila P. Burakova, Natalya E. Karuzina, Bogdan S. Melnik, Valentina A. Kratasyuk

**Affiliations:** 1 Siberian Federal University, 660041 Krasnoyarsk, Russia; dgulnov@sfu-kras.ru (D.V.G.); marina_2506@mail.ru (M.A.G.); lsukovatyy@sfu-kras.ru (L.A.S.); burakoval@mail.ru (L.P.B.); nkaruzina@sfu-kras.ru (N.E.K.); vkratasyuk@sfu-kras.ru (V.A.K.); 2 Photobiology Laboratory, Institute of Biophysics SB RAS, 660036 Krasnoyarsk, Russia; 3 Institute of Protein Research, Russian Academy of Sciences, 142290 Pushchino, Russia; bmelnik@phys.protres.ru

**Keywords:** bacterial luciferase, urea-induced denaturation, time-resolved spectroscopy, conformational stability, FRET, tryptophan fluorescence, molecular dynamics, unfolding pathway

## Abstract

Detecting the folding/unfolding pathways of biological macromolecules is one of the urgent problems of molecular biophysics. The unfolding of bacterial luciferase from *Vibrio harveyi* is well-studied, unlike that of *Photobacterium leiognathi*, despite the fact that both of them are actively used as a reporter system. The aim of this study was to compare the conformational transitions of these luciferases from two different protein subfamilies during equilibrium unfolding with urea. Intrinsic steady-state and time-resolved fluorescence spectra and circular dichroism spectra were used to determine the stages of the protein unfolding. Molecular dynamics methods were applied to find the differences in the surroundings of tryptophans in both luciferases. We found that the unfolding pathway is the same for the studied luciferases. However, the results obtained indicate more stable tertiary and secondary structures of *P. leiognathi* luciferase as compared to enzyme from *V. harveyi* during the last stage of denaturation, including the unfolding of individual subunits. The distinctions in fluorescence of the two proteins are associated with differences in the structure of the C-terminal domain of α-subunits, which causes different quenching of tryptophan emissions. The time-resolved fluorescence technique proved to be a more effective method for studying protein unfolding than steady-state methods.

## 1. Introduction

The conformational stability of proteins and the methods of its evaluation have been the subject of intense interest for researchers from various fields, including molecular and cellular biophysics, biochemistry, food chemistry, biomedicine and many others. The disturbance of protein stability due to gene mutations or the influence of external physical and chemical factors can lead to the appearance of misfolded species or structural intermediates which cause various disease states in cells and tissues [1,2]. Fluorescence spectroscopy techniques are widely used to probe the conformational transitions of proteins [3]. The analysis of the time-resolved protein fluorescence has several advantages over other optical steady-state techniques; for example, fluorescence lifetimes do not depend on protein concentration, which is especially important for in vivo studies [4]. Meanwhile, there is a downside to the use of tryptophan residues as natural fluorophores to monitor the conformational states of proteins. Namely, it seems difficult to compare the unfolding pathways of two proteins bearing different numbers of tryptophans, which are located in different parts of the protein globule. An additional difficulty arises from the lack of understanding of the nature of the components of the tryptophan fluorescence lifetime. In the current study, we investigated whether time-resolved fluorescence can be used to detect differences in conformational stability under the urea-induced unfolding of two highly homologous multi-tryptophan proteins—bacterial luciferases. Molecular modelling methods were utilized to confirm that the observed differences are not caused by the distinct locations of tryptophans within the protein globules.

Luciferases catalyze specific reactions with the formation of a product in an electronically excited state; this product deactivates with the emission of light within the visible range of the spectrum. These chemical reactions ensure bioluminescence in living organisms, which serves various important functions. Bacterial luciferase catalyzes the oxidation of reduced flavin mononucleotides and long chain aliphatic aldehydes accompanied by the generation of blue-greenish light [5]. This enzymatic reaction is widely used as a reporter system in different fields, including molecular biology [6], environmental monitoring [7] and medical diagnostics [8].

Phylogenetic analysis of luciferase amino acid sequences have revealed that all known bacterial luciferases can be divided into two subfamilies [9], which are conditionally called “fast” and “slow” enzymes based on the kinetic peculiarities of their reactions [10]. Historically, during the last several decades, the bulk of research on bacterial bioluminescent reaction has been conducted using *Vibrio harveyi* luciferase, belonging to the “slow” enzymes. Those studies were concerned with the mechanism of catalysis [11], the structure [12,13], the folding and unfolding processes [14], the structure-function relationship [15], and other aspects. Meanwhile, the evident division of the luciferase sequences into two groups with high homology within each group suggests that the subfamilies could differ not only in their functional properties, but also in conformational stability. The published data do not allow us to confirm or refute this assumption, since luciferase unfolding has never been studied for at least two enzymes from different phylogenetic groups under the same conditions. In this work, we attempted to fill this gap by analyzing the conformational transitions of the “fast” *P. leiognathi* luciferase and the “slow” *V. harveyi* luciferase.

**Scheme 1:**
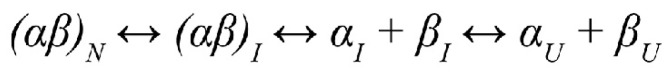
Four-state mechanism of *V. harveyi* luciferase unfolding with urea. *(αβ)_N_*–luciferase heterodimer in the native state, *(αβ)_I_*–inactive dimeric intermediate, *α_I_* and *β_I_*–dissociated subunits in the folded state, *α_U_* and *β_U_*–unfolded subunits.

Structurally, bacterial luciferase is composed of two non-identical subunits (*α* and *β*), both of which assume the (β/α)_8_ barrel fold [12]. Apart from the high similarity of the secondary and tertiary structures, the α- and β-subunits have a high sequence homology, especially at the N-terminal, that reflects their possible origin from the same gene as a result of duplication [16]. However, the *α*-subunit contains the active site and is larger due to the insertion of a mobile loop playing an important role in binding substrates and shielding reaction intermediates in the active site [15,17]. The dimerization of luciferase involves forming 22 hydrogen bonds between conserved amino acids of the subunits [12,18]. It is noteworthy that mutation of these amino acids leads not only to dissociation of the heterodimer, but in some cases to conformational changes in the α-subunit (including the structure of the active site) [13,19,20]. Thorough study of the denaturation of *V. harveyi* luciferase with urea led to the conclusion about the four-state mechanism of unfolding, shown in Scheme 1 [20].

The first intermediate *(αβ)_I_* was found to populate at about 2 M of urea, while folded dissociated subunits *α_I_* and *β_I_* populated at 3.5 M of urea [14].

The aim of this study was to compare the conformational transitions of “fast” *P. leiognathi* and “slow” *V. harveyi* luciferases during equilibrium unfolding with urea. To detect the structural transitions of the proteins, we used optical techniques, including steady-state and time-resolved fluorescence spectroscopy and circular dichroism spectroscopy. In addition to information on the stability of the two homologous proteins, we attempted to clarify the question of whether the tryptophan fluorescence lifetime of bacterial luciferase carries new information about the unfolding pathway as compared to widely used steady-state methods. In previous studies, we established that the analysis of fluorescence lifetime allowed for the resolution of two distinct transitions of bovine carboanhydrase II during equilibrium unfolding with urea, which was impossible using other non-kinetic techniques [21]. One more issue that we endeavored to address is the dependence of conformational stability parameters obtained using fluorescence spectroscopy on the number of the tryptophan residues contained in the protein. The studied proteins have different numbers of tryptophans in their structures: *P. leiognathi* luciferase contains seven (*α*W40, *α*W182, *α*W194, *α*W250, *α*W277, *β*W182, *β*W194), while *V. harveyi* contains eight (the same plus *α*W131).

## 2. Results

### 2.1. Fluorescence Lifetime Change during Urea-Induced Unfolding of Luciferases

**Figure 1:**
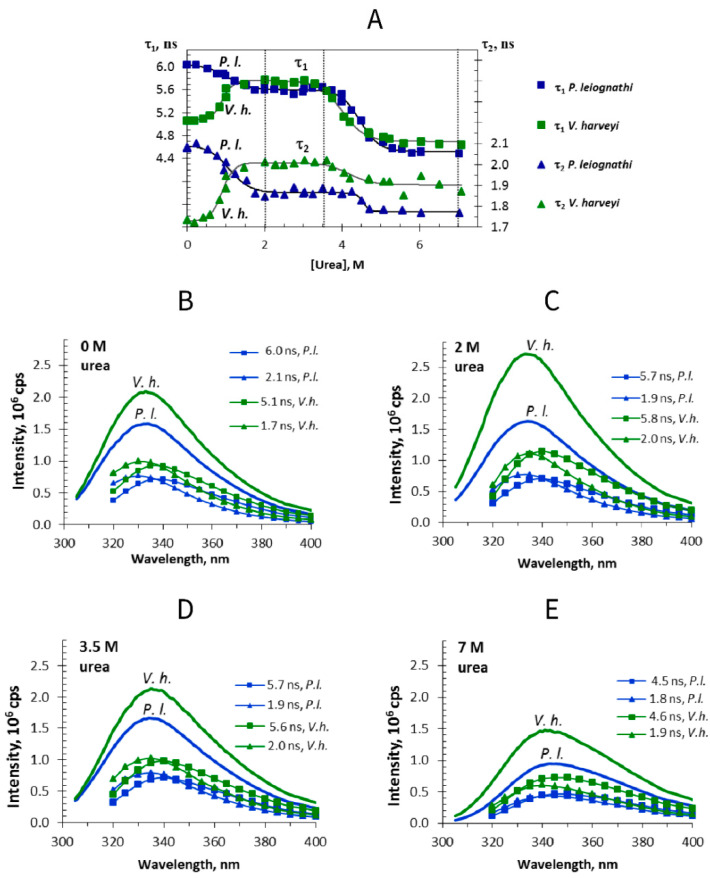
Dependence of the fluorescence lifetime components τ_1_ (squares) and τ_2_ (triangles) on urea concentration for *P. leiognathi* (blue) and *V. harveyi* (green) luciferases (**A**), and the steady-state fluorescence spectra of native (**B**), intermediate (**C**,**D**) and unfolded (**E**) states of luciferases (excitation at 296 nm, protein concentration is 1.7 μM). In panel (**A**), vertical dotted lines refer to the urea concentrations at which the fluorescence spectra are shown in panels (**C**–**E**); solid lines represent fitting to a double Boltzmann function. By marked lines in panels (**B**–**E**) the decay-associated spectra based on Equation (2) are shown for the lifetime components.

We measured the fluorescence decays under excitation of 296 nm for *V. harveyi* and *P. leiognathi* luciferases in buffer and after incubation at various urea concentrations. The global analysis of the decays in a wide emission range (305–410 nm) made it possible to determine not only lifetimes, but also their contributions to the overall fluorescence, which allowed the decay-associated spectra of the proteins to be obtained. The deconvolution procedure revealed 3 lifetime components: 4.5–6.0 ns (τ_1_), 1.7–2.1 ns (τ_2_), and 0.03–0.2 ns (τ_3_) for each sample. The components τ_1_ and τ_2_ together made the major contribution to the protein fluorescence of all samples (about 90%), and their values could be reliably resolved by the instrument, unlike the value of τ_3_. That is why the τ_3_ change during protein unfolding is not discussed in the current investigation. The dependence of τ_1_ and τ_2_ on the urea concentration is shown in Figure 1A, demonstrating a similar change of the two lifetime components for each luciferase. At >3.5–4 M of urea, τ_1_ and τ_2_ decrease for both proteins. However, at low urea concentrations, contrasting patterns were revealed for both studied luciferases: for *V. harveyi,* an increase in the lifetimes was observed up to 1.5 M, whereas for *P. leiognathi* luciferase, a decrease in the lifetimes was detected in the concentration range of 0–2 M. On the whole, the change in fluorescence lifetimes with increasing urea concentrations reflects two structural transitions for each luciferase. From the previous study of *V. harveyi* luciferase unfolding, it was concluded that two intermediates of this protein populated at about 2 M and 3.5 M [20] of urea. A comparison of the spectral properties of these intermediates, as well as the native and unfolded states of two luciferases, are presented in Figure 1B–E. One can see that: (i) *V. harveyi* luciferase demonstrates a higher intensity of fluorescence at the same concentration of urea (Figure 1B–E), which was as expected due to the extra tryptophan residue in its structure as compared to *P. leiognathi* luciferase; (ii) for *V. harveyi* luciferase, the fluorescence lifetime increase in the range 0–2 M of urea is accompanied by a fluorescence intensity increase (Figure 1B,C); (iii) the two lifetime components τ_1_ and τ_2_ have similar spectral fractions in protein fluorescence at all urea concentrations. It is noteworthy that the spectral fractions of the two fluorescence lifetimes were found to remain nearly the same at all studied urea concentrations (Figure S1).

**Figure 2:**
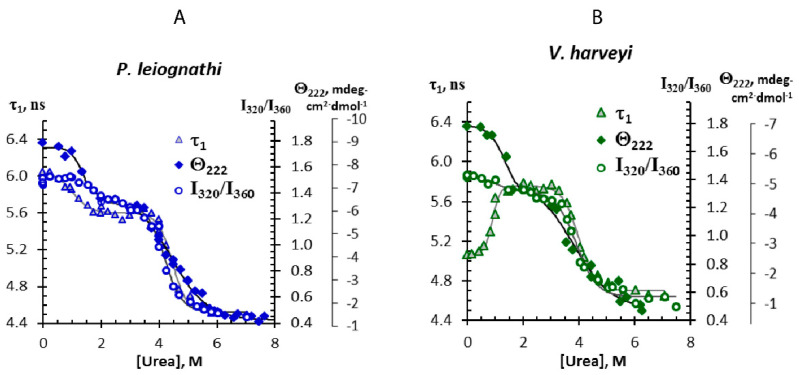
Comparison of the optical parameter changes in equilibrium unfolding of *P. leiognathi* (**A**) and *V. harveyi* (**B**) luciferases obtained by different methods: the ratio I_320_/I_360_ (**◯**), molar ellipticity at 222 nm, Θ_222_, (◇) and the fluorescence lifetime component τ_1_ (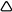). Solid lines represent fitting to a double Boltzmann function.

Additionally, we obtained the transition curves of luciferases using the other optical parameters: the molar ellipticity at 222 nm in the circular dichroism spectra (Θ_222_), and the ratio of intensities at 320 and 360 nm in the steady-state fluorescence spectra (I_320_/I_360_). A comparison of the parameters obtained by different methods is shown in Figure 2. One can see that variation of Θ_222_ reveals two transitions similar to those detected by the fluorescence lifetime component τ_1_, whereas the change of I_320_/I_360_ is not so pronounced for the urea concentrations <2 M, especially for *V. harveyi* luciferase. The entire transition curves were fitted by the double Boltzmann function (see the Section 4) (Figure 2).

The fitted transitions detected by different methods allowed thermodynamic parameters to be calculated and, hence, the structural stability of the two luciferases to be compared (Table 1).

The observed difference in fluorescence characteristics of the two proteins during unfolding could have been caused by the distinguished location of tryptophan residues in the protein globule and/or the different microenvironment of these fluorophores. To check this relation, we analyzed the structural and dynamic properties of the two luciferases using the molecular modeling technique.

### 2.2. Tryptophans of the Bacterial Luciferases as Fluorescent Reporters

To compare the position of tryptophan residues and the properties of their microenvironment in the two luciferases, molecular dynamics simulations of the proteins were performed for 40 ns. The tertiary structure of *P. leiognathi* luciferase was modeled for the first time (see details in the Section 4). We found that seven tryptophans of *P. leiognathi* luciferase are located in approximately the same position as compared to *V. harveyi* luciferase, in spite of a slight difference in the total sequence lengths of the proteins. The relative positions of those tryptophans in the luciferases after the tertiary structure alignment are presented in Figure S2. Instead of αW131 presented in *V. harveyi* luciferase, the *P. leiognathi* structure contains a phenylalanine residue in the same position (Figure S2). The position of *α*W277 (located in the mobile loop of the luciferases) revealed the most prominent difference between the proteins (Figure S2).

For each tryptophan, the microenvironment properties, which are known to influence the fluorescence characteristics of this residue, were determined [22]. In particular, the analyzed parameters include the total number of protein atoms within 7 Å of any tryptophan atom (N_Σ_) and the number of polar atoms among them (N_pol_), the solvent-accessible surface area (SASA), and others. All the calculated characteristics are summarized in Table 2.

Thus, a packing density of the tryptophans (quantified by N_Σ_) is higher in *V. harveyi* luciferase, and the most buried ones are *α*W250 and *α*W277. However, the apparent polarity of the tryptophan microenvironments (as indicated by N_pol_) does not differ significantly between the two luciferases.

The majority of the tryptophan residues in the luciferases demonstrate a small solvent-accessible surface area (0–17% from the total surface area), i.e., the residues are buried in the protein globule. The exceptions are the *α*W277 of both proteins, with about 40% of the surface area exposed to the solvent (Table 2). It is noteworthy that *α*W277 also has the most flexible microenvironment in the studied structures, because a significant variation of its SASA values was observed during molecular dynamics simulations.

The first step of luciferase unfolding includes denaturation of the C-terminal domain of the protein (236–355 amino acid residues) [23]. To estimate the sensitivity of tryptophan fluorescence at this step, the number of the luciferase C-terminal domain atoms neighboring each tryptophan was also counted (N_C-term_, Table 2). The highest value of N_C-term_ was found for *α*W194 and *α*W277. Similarly, it was revealed that changes in the fluorescence spectra of any tryptophan (except for *α*W277 of *V. harveyi*) can be seen in response to the secondary structure disruption, because their microenvironment mainly consists of the amino acids incorporated into α-helixes or β-strands (>54% of atoms) (N_sec_, Table 2).

The second stage of luciferase unfolding involves subunit dissociation. Among all tryptophans, only *α*W277 of *P. leiognathi* luciferase was found to have the atoms of a *β*-subunit in its microenvironment within 7 Å distance from the side chain atoms (N_β-sub_, Table 2).

**Figure 3:**
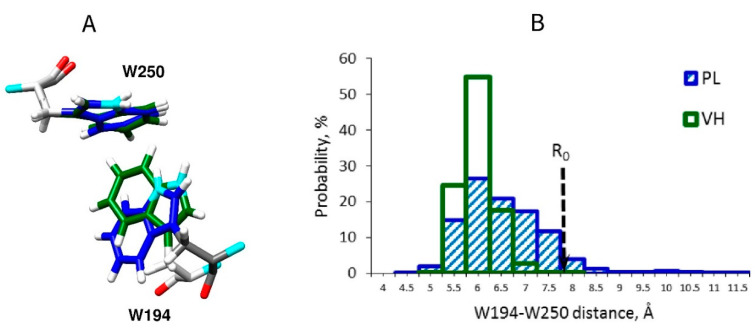
(**A**) Relative position of *α*W250 (top) and *α*W194 (bottom) in the structures of *P. leiognathi* (blue) and *V. harveyi* (green) luciferases. The protein structures after molecular dynamics simulations were aligned so that the positions of *α*W250 coincided. The corresponding FRET efficiencies between ^1^La states are 86% (*P. leiognathi*) and 82% (*V. harveyi*). (**B**) The distribution of distances between *α*W250 and *α*W194 during molecular dynamics simulations of luciferases *P. leiognathi* (blue) and *V. harveyi* (green). The black arrow indicates the Förster radius (R_0_) for FRET between the tryptophans [26].

The Förster resonance energy transfer (FRET) between nearby tryptophans can also have a strong effect on the protein fluorescence [24]; therefore, we performed a search for potential donor-acceptor pairs among the tryptophans of the luciferases. From distances *R*, it was concluded that FRET can occur only between *α*W194 and *α*W250. The aligned position of these tryptophan pairs in the two luciferases is presented in Figure 3A. Apparently, the donor-acceptor orientation is not identical for the two proteins. Using atom coordinates, we calculated the orientation factor κ^2^ from Equation (5) and the FRET efficiency *E* from Equation (4) between ^1^L_a_ states of the tryptophans presented in Figure 3A. As a result, the value of κ^2^ was 0.32 and 0.82, and the efficiency was 86% and 82% for luciferases from *P. leiognathi* and *V. harveyi*, correspondingly. Notably, the observed less favorable orientation of the *V. harveyi* donor-acceptor pair is compensated by a shorter distance between the tryptophans. Taking into account the intrinsic dynamics of the proteins, we analyzed the distribution of the distances between *α*W194 and *α*W250 during molecular dynamics simulations (Figure 3B). One can see that *V. harveyi* luciferase demonstrates less structural mobility: most of the time its tryptophans are separated by the intervals of 5.5–6.5 Å, whereas in *P. leiognathi* luciferase, the donor-acceptor distance varies in a wider range of 5.0–8.5 Å. This finding correlates with the higher packing density of *α*W250 in *V. harveyi* luciferase (N_Σ_, Table 2), which could have been preventing sidechain mobility. The observed distances for both proteins are shorter than the Förster radius for the Trp-Trp pair (7.8 Å), what means that the FRET efficiency could have been higher than 50% (Figure 3B). However, in the study of the mutants of the *Photorhabdus luminescens* luciferase (a close homologue of *V. harveyi* luciferase), it was concluded that for the donor-acceptor pair *α*W194-*α*W250, the range of FRET is restricted to a few angstroms. [25]. In general, our analysis indicates that the resonance energy transfer between *α*W194 and *α*W250 could have a stronger effect on the intrinsic fluorescence of *V. harveyi* luciferase than on that of *P. leiognathi* luciferase because of a shorter mean distance and lower protein dynamics. It should be noted that such short distances between tryptophans also do not allow us to exclude other mechanisms of energy transfer, such as electron transfer or charge transfer, which begin to be observed at <10 Å distances [26].

An additional mechanism of change in the fluorescence intensity and the lifetime of tryptophans in a protein is quenching by the neighboring atoms of both amino acid side chains and the protein backbone chain [27]. Careful examination of the atoms surrounding the tryptophans revealed three differences between the luciferases: (i) the distance between the C_ε3_ atom and the carbonyl group of *α*W182 in the *V. harveyi* protein is shorter than in the *P. leiognathi* protein (3.6 and 4.5 Å, respectively); (ii) *α*D120, known for its ability to quench tryptophan fluorescence with its side chain, is located 4 Å away from *α*W277 in *V. harveyi*, whereas in *P. leiognathi* luciferase all potential quenchers of *α*W277 are at distances >5.5 Å; (iii) the *α*W131 of *V. harveyi*, which has no analogue in the *P. leiognathi* structure, resides near the sulfur atom of *α*C130 (4.4 Å), and its fluorescence can be also quenched. Notably, for *Photorhabdus luminescens* luciferase, the possibility of the *α*W182 fluorescence being quenched by the protein groups was revealed earlier [25]. Thus, a more pronounced effect of quenching on the tryptophan fluorescence could be expected in *V. harveyi* luciferase than in *P. leiognathi* luciferase.

## 3. Discussion

### 3.1. The Origin of the Difference in Tryptophan Fluorescence of the Two Luciferases

This study was aimed at comparing the structural stability of two highly homologous proteins—luciferases from bacteria *P. leiognathi* and *V. harveyi—*during urea-induced unfolding. For this, a detailed analysis of fluorescence properties, including the time-resolved properties, was first performed for the two luciferases after incubation at various urea concentrations. This study allowed us to identify features that differ for the proteins under consideration, despite the great similarities of their structures.

First, *V. harveyi* luciferase in its native state demonstrates a higher fluorescence intensity per one protein molecule, but a shorter fluorescence lifetime, than *P. leiognathi* luciferase (Figure 1B). Generally, a decrease in the fluorescence lifetime of a fluorophore indicates enhancement of the nonradiative pathways of the relaxation of the excitation energy (i.e., electron/proton/energy transfer, internal conversion and others) and correlates with a lower fluorescence quantum yield [27]. For tryptophan fluorescence in proteins, the following mechanisms of quenching are recognized [28]: (a) proton transfer from nearby charged amino groups, which is effective from lysine and tyrosine side chains; (b) electron transfer quenching by disulfides and amides or protonated carboxyl groups in which cysteine, histidine, glutamine, asparagine, as well as aspartic and glutamic acids, could take part; (c) electron transfer quenching by peptide bonds in the protein backbone; and (d) resonance energy transfer among the tryptophan residues.

We obtained data indicating that the shorter τ_1_ in the native state of *V. harveyi* luciferase compared to that of *P. leiognathi* luciferase (5.1 vs. 6.0 ns) can be the result of a more effective FRET between *α*W194 and *α*W250 (mechanism (d)), facilitated by a closer spacing between these tryptophans (Figure 3) and by a higher packing density (N_Σ_, Table 2). Additionally, examination of the tryptophan microenvironments revealed that in *V. harveyi* luciferase, the fluorescence of *α*W182 can be quenched by its carbonyl group (mechanism (c)) and the fluorescence of *α*W277 can be quenched by *α*D120 (mechanism (b)), which is not the case for the *P. leiognathi* enzyme. Additionally, the presence of one more tryptophan (*α*W131) in the structure of the *V. harveyi* protein probably compensates for the loss of intensity due to fluorescence quenching, which explains the contradiction between the lifetimes and the intensities observed for the luciferases in their native state (Figure 1B). The identification of more precise mechanisms seems difficult in the scope of the current work because mutations of the tryptophan residues are required to estimate their contribution to the intensity and the lifetime components of the protein fluorescence.

Second, the fluorescence lifetimes of the two luciferases change in opposite ways during the first stage of denaturation (at <2 M of urea): for *V. harveyi* luciferase, τ_1_ and τ_2_ show an increase, while for the *P. leiognathi* enzyme, the lifetime components decrease (Figure 1A–C). This stage includes the unfolding of the C-terminal domain of the luciferase *α*-subunit (236–355 a.r.) that was shown in different studies [23]. Thus, the increase of fluorescence intensity and the lifetimes τ_1_ and τ_2_ of *V. harveyi* luciferase at low urea concentrations could be the result of neutralizing the quenching effects of the microenvironment on the tryptophans discussed above. Indeed, *α*W277 and *α*W250 themselves are localized on the C-terminal domain, and in close proximity to *α*W194, 43% of all atoms belong to this domain (N_C-term_, Table 2). In turn, the small decrease of τ_1_ and τ_2_ of *P. leiognathi* luciferase during the first stage of unfolding can originate from the disordering of the *α*W277 microenvironment. According to the analysis, this tryptophan is located among the residues included in the β-strands (N_sec_, Table 2), which could cause a higher quantum yield due to more ordered and less mobile surroundings. In this case, the unfolding of the C-terminal domain would result in a decrease of the fluorescence lifetime. It is noteworthy that the two luciferases in the intermediate state (αβ)*_I_* (at about 2 M of urea) acquire almost identical fluorescence properties (Figure 1A,C). This means that all the differences observed in the native state are associated with the structural features of the C-terminal domain of the two luciferases. After the unfolding of this domain, the luciferases become almost indistinguishable by fluorescence spectroscopy.

The next denaturation stage is the subunit dissociation. This does not influence the optical characteristics of either of the luciferases, which is evident from a slight change in various parameters in the range of 2–3.5 M urea (Figure 1A,C,D and Figure 2). This result is not surprising, since all the tryptophans, with the exception of *α*W277 of *P. leiognathi* luciferase, are located far from the intersubunit interface (N_β_, Table 2).

The last stage of denaturation is the unfolding of the subunits. This occurs in the same way for the two proteins (Figure 1A,D,E and Figure 2) with the only difference seen in the fluorescence intensity, which is associated with the presence of the additional *α*W131 in *V. harveyi* luciferase.

Probably due to the unidirectional change of the lifetimes τ_1_ and τ_2_ during denaturation, their relative contributions to the protein fluorescence do not change and remain at about 45 and 55%, respectively (Figure 1 B–E and Figure S1).

Thus, the structural differences between *P. leiognathi* and *V. harveyi* luciferases affect the fluorescent properties of these highly homologous proteins only in the native state when the globules are the most densely packed.

### 3.2. Comparison of the Stability of the Luciferases Structures

**Figure 4:**
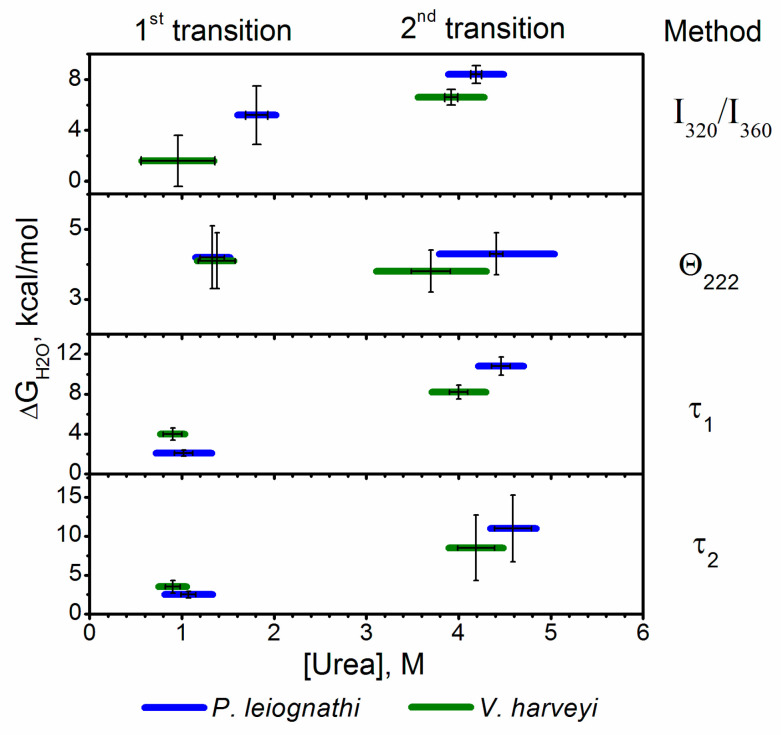
The urea concentration ranges of the structural transitions for the luciferases from *V. harveyi* (green) and *P. leiognathi* (blue) and corresponding ΔG_H2O_, as determined by different techniques (Table 1). The length of the sticks reflects the transition width k_i_ in accordance with the parameters obtained by fitting with a Boltzmann function; the middle of the sticks is the transition midpoint [Urea]_50%_.

To reveal the unfolding stages of the two luciferases, we used time-resolved fluorescence spectroscopy in combination with other traditional techniques (circular dichroism spectroscopy and steady-state fluorescence). Measuring various optical characteristics of the proteins at different urea concentrations made it possible to determine the thermodynamic parameters of the unfolding luciferases (Table 1) and to plot a diagram of the relative position of the transitions during denaturation with the corresponding thermodynamic characteristic ΔG_H2O_ (Figure 4).

As seen from Figure 4, the transitions of the luciferases demonstrated a similar width, except for the first transition determined by τ_1_. The difference in the transition widths and midpoints causes the obtained relationship between the free energy changes (ΔG_H2O_, Table 1) for the two luciferases. In spite of the shift in the transition midpoint for the higher urea concentration, *P. leiognathi* luciferase displayed a lower ΔG_H2O_ during the first transition, determined by τ_1_. The change in the time-resolved characteristics of the protein fluorescence points out that the *P. leiognathi* luciferase has a more stable tertiary and secondary structure than the *V. harveyi* luciferase, i.e., the transitions have higher energetic barriers (τ_1_, Figure 4). Of note, the opposite result obtained by τ_1_ for the initial denaturation stage is in a good agreement with the conclusions made in [29]. Based on the thermal inactivation of the luciferases from different bacterial species, Holzman et al. observed that the apparent melting point of the *V. harveyi* luciferase is at least 10 °C higher than that of the *P. phosphoreum* luciferase (which is of the same subfamily with the *P. leiognathi* protein). This study seems to be the only one which is concerned with the indirect comparison of structural stability of the luciferases from the different bacterial species. Later, it was shown that the urea-induced loss of enzymatic activity of the *V. harveyi* luciferase occurs with a midpoint at about 1.6 M of urea [30], indicating that it corresponds to the first structural transition observed with spectroscopic methods.

We found that the secondary structure of the *V. harveyi* luciferase demonstrates lower stability (Θ_222_, Figure 4) than the *P. leiognathi* luciferase. According to the Θ_222_ change, the second visible transitions start at 3.0 and 3.8 M of urea for *V. harveyi* and *P. leiognathi* luciferase, respectively. The structural origins for this difference are discussed below.

Finally, considering our results in terms of the protein unfolding pathway (as the number and the structure of the intermediate species), we can conclude that the unfolding pathway is the same for the studied luciferases. It is an important result because, in some cases, different unfolding pathways have been confirmed for highly homologous proteins (for example, alpha amylases with 88% sequence similarity [31]).

### 3.3. The Origin of the Difference in Stability of Two Luciferases

**Figure 5:**
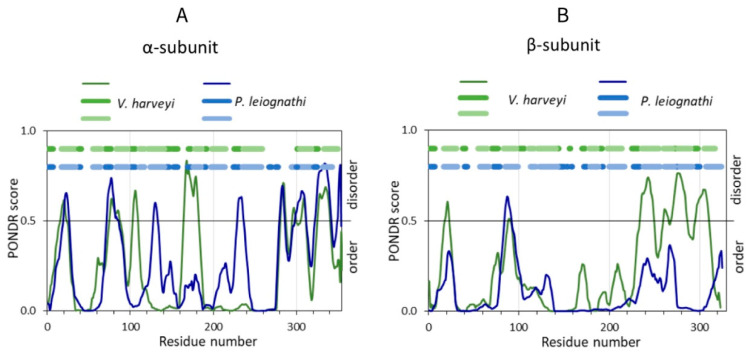
Intrinsic disorder propensities for amino acid sequences of the *α*-subunit (**A**) and *β*-subunit (**B**) of the luciferases *V. harveyi* (green lines) and *P. leiognathi* (blue lines), as predicted with PONDR FIT [33]. Horizontal thick lines refer to the position of the amino acids, which are included in an α-helix (lighter color) or β-strand (darker color). Distribution of secondary structure segments in polypeptide chains was retrieved using UCSF Chimera software.

To identify the regions within the luciferase structures which could demonstrate less conformational stability, we used algorithms predicting intrinsically disordered regions relative to the approach described in [32]. This approach is based on the utilization of computational tools for the per-residue evaluation of the intrinsic disorder predisposition to search for the “weakest spot” of a query protein. We used PONDR VLXT software [33] to predict the tendency of certain regions of a polypeptide chain of the luciferases to be either structured or intrinsically disordered based on statistics or properties of amino acid residues. The analysis revealed that the *α*-subunits of *V. harveyi* and *P. leiognathi* luciferases demonstrate very similar properties: about 22% of their amino acid residuals (a.r.) were predicted as located in disordered parts within 8 regions, with the longest one of 18 a.r. (Figure 5). However, for the *β*-subunits different patterns were obtained: for the *V. harveyi* luciferase about 20% of a.r. were predicted to be disordered, whereas for the *P. leiognathi* luciferase only about 3% of those a.r. were found. The large region of 227–325 a.r. of the *β*-subunit demonstrates different predisposition to be disordered for the *V. harveyi* and *P. leiognathi* luciferase. In the crystal structure of the luciferase [13], the α-helixes and β-strands were resolved in this region. The apparent contradiction could mean that this part of the subunit requires interaction with the remaining rigidly packed part of the protein to obtain the correct three-dimensional structure [32]. Thus, our analysis indicates that the observed lower stability of the secondary structure of the *V. harveyi* luciferase in comparison with the *P. leiognathi* enzyme could be attributed to the difference in the sequence of the C-terminal part of the β-subunit. The large width of the second transition, revealed by the CD technique for the *V. harveyi* luciferase (Θ_222_, Figure 4), could reflect the unfolding of the β-subunit first, which occurs at lower urea concentrations than for the *P. leiognathi* enzyme. Moreover, no tryptophan residues are located in the presumably weakened region of the luciferase β-subunit, so the fluorescent signal is not sensitive to the initial stage of the second transition, in contrast to the circular dichroism signal (Figure 4).

### 3.4. Informativeness of Different Experimental Techniques on Luciferase Unfolding

The results obtained in this work made it possible not only to compare the stability of the secondary and tertiary structures of the closely related proteins, but also to evaluate the sensitivity of different experimental methods to individual stages of protein unfolding. Thus, the first stage of the unfolding of both luciferases has little effect on the position of their fluorescence spectrum (I_320_/I_360_). This parameter changes by less than 20%, while the shifts in other optical parameters are more pronounced, as can be seen from the values of the fraction in Table 1. This can be explained by the fact that the unfolding of the C-terminal domain of proteins is probably not accompanied by a significant change in the polarity of the environment of the majority of the tryptophan residues. The penetration of water into the deeper parts of the luciferases occurs during the next unfolding stages. Thus, in this study, the time-resolved fluorescence demonstrates obvious advantages and detects the subtle initial structural transitions of the proteins, which cannot be seen from the steady-state emission spectra.

It also turned out to be impossible to reliably obtain the characteristics of the second transition of the luciferases with a midpoint of about 4 M of urea using lifetime component τ_2_. The amplitude of the whole change of τ_2_ during this unfolding is only ~0.1 ns, and the resolution of the instrument does not allow for accurate determination of the gradual shift of this lifetime component.

For the fluorescence of bacterial luciferase, lifetime components τ_1_ and τ_2_ were found to reflect similar information about the stages of protein unfolding (Table 1). It was previously shown that this is not the case for some proteins (see the example of bovine carboanhydrase II [21]). It should be emphasized that the structural and/or dynamic reasons for the similarities or differences in information based on the fluorescence lifetimes of the protein are not yet understood. The study of denaturation of various proteins containing a single tryptophan in their structure could shed light on this problem. The recent findings point out the importance of the distribution of electrostatic potential around tryptophan in modulating the probability of the formation of a charge transfer state after photon absorption, which, in turn, influences the quantum yield and the lifetime of the tryptophan fluorescence [34]. However, an unambiguous and available method for analyzing this structural characteristic for a specific protein has not yet been developed.

## 4. Materials and Methods

### 4.1. Materials

Lyophilized recombinant luciferase from *Photobacterium leiognathi* (99% purity) was purchased from Biolumdiagnostika Ltd. (Krasnoyarsk, Russia). Urea (Panreac, Germany), Na_2_HPO_4_ and KH_2_PO_4_ (AppliChem, Germany) were used without additional purification. All solutions were prepared using Na_2_HPO_4_/KH_2_PO_4_ buffer (0.05 M, pH 6.9). The concentration of urea (0.25–8 M) was determined using a laboratory refractometer, IRF-454 B2M (Kazan Optical and Mechanical Plant, Russia).

### 4.2. Expression and Purification of V. harveyi luciferase

The pET19b-VhLuxAB plasmid containing the coding part of the *V. harveyi* (MAV strain) luxAB gene was expressed in *E. coli* strain BL21 (DE3) via Codon Plus RIPL (Stratagene, USA). The cells harboring the plasmid were grown in the LB medium containing 200 μg/mL ampicillin at 37 °C, and IPTG was added to the final concentration of 0.25 mM when the cell density reached an OD_600_ of 0.6–0.7. Following the induction with IPTG, the cell culture was allowed to grow for 18 h at 23 °C. With this expression, luciferase mostly accumulated in the cytoplasm in a soluble form. Cells were harvested, centrifuged, and resuspended in 5 mL× *g* of 20 mM Tris-HCl, pH 7.0 before lysis by ultrasonication. After lysis, the cell debris was removed by centrifugation, and the supernatant containing the extracted luciferase was purified with ion-exchange chromatography on a HiTrap Q HP 5 column (GE Healthcare, USA) with a NaCl linear gradient (0–1 M). The portion of the eluate containing luciferase was concentrated, diluted 25 times in 50 mM phosphate buffer, pH 7.0, and sedimented with ammonium sulfate (25.8 g per every 50 mL of the solution). After centrifugation the precipitate was diluted in 50 mM phosphate buffer, pH 7.0, with 0.15 M NaCl and purified with gel filtration on a Superdex 75 (GE Healthcare, USA). Protein concentrations were first estimated using the DC Protein Assay Kit with bovine serum albumin as a standard (Bio-Rad, USA). More accurate protein concentration was determined spectrophotometrically using the protein extinction coefficient calculated by the method of Gill and von Hippel [35].

To confirm the proper luciferase folding, its functionality was tested by the flavin-injection technique [36].

### 4.3. Steady-State Fluorescence

The spectral characteristics of the luciferases at concentration of 1–2 μM were measured after 18 h of incubation with urea (0.25–8 M) at room temperature. The measurements were carried out at 25 °C unless otherwise specified.

The absorption spectra were measured using a Cary 5000 spectrophotometer (Agilent Technologies, Australia) with an integrated Pelletier temperature controller. Steady-state fluorescence spectra of the protein were measured with a Fluorolog-3 spectrofluorometer (Horiba Jobin Yvon, USA). The intensity was collected within the range of 305–450 nm under excitation at 296 nm. All fluorescence spectra were corrected for PMT spectral sensitivity, inner filter effect and background signal [28].

The spectral shift of fluorescence was described in terms of the ratio of the intensities at 320 and 360 nm, I_320_/I_360_.

### 4.4. Time-Resolved Fluorescence

Time-resolved fluorescence decays were obtained using a time-correlated single photon counting (TCSPC) DeltaHub module of Fluorolog-3 (Horiba Jobin Yvon, USA). A NanoLED N-295 with the peak wavelength at 296 nm and pulse duration of <1.2 ns was applied for excitation. Emission was detected within the range of 320–410 nm with a step of 5 nm; time resolution was 0.027 ns/channel.

Time-resolved fluorescence experiments were carried out using the TCSPC technique [37]. The fluorescence lifetime profile of the protein consisting of a sum of three exponentials with lifetimes *τ_i_* and amplitudes α*_i_* was deconvoluted from the observed fluorescence decay *I*(*t*):

(1) $$I\left(t\right)=S\left(t\right)⊗\sum_{i=1}^{N}\alpha_{i}e^{-t/\tau_{i}}$$

where *S*(*t*) is the instrumental response function measured with a highly scattering solution, Ludox. The fit quality was evaluated by its global χ^2^ value and weighted residuals.

The decay-associated spectra of fluorescence were calculated as follows:

(2) $$I_{i}^{\lambda }=f_{i}^{\lambda }I_{ss}$$

where *I_ss_* is the steady-state fluorescence spectra, and $f_{i}^{\lambda }$ is the contribution of each lifetime component to fluorescence at a certain wavelength λ:

(3) $$f_{i}^{\lambda }=\frac{\alpha_{i}^{\lambda }\tau_{i}}{\sum_{j=1}^{N}\alpha_{j}^{\lambda }\tau_{j}}$$

where $\alpha_{i}^{\lambda }$ is the amplitude of the *i*-component of the lifetime at a wavelength λ. The total spectral contribution of the lifetime component *τ_i_* to protein fluorescence, *f_i_*, was calculated as the average of the $f_{i}^{\lambda }$ values.

### 4.5. Circular Dichroism Spectroscopy

The CD spectra were obtained using Jasco-810 and a Jasco-1500 spectropolarimeters (Jasco, Japan). The far-UV CD spectra were recorded for *P. leiognathi* luciferase in a 1 mm path length cell in the range of 190–260 nm, with a step size of 0.2 nm, and for *V. harveyi* luciferase in a 0.1 mm path length cell in the range of 190–250 nm, with a step size of 1 nm. At least two scans were averaged for all spectra. Spectra were baseline corrected. The measurements were performed at room temperature.

### 4.6. Data Treatment

The urea-induced transition curves obtained from the change of the optical parameters were fitted by the double-Boltzmann function, available in the Origin 8.0 software (OriginLab Corp.). The transition width, k_i_, and the transition midpoints, [Urea]_50%_, were obtained to compare the structural stability of the proteins.

### 4.7. Förster Resonance Energy Transfer (FRET)

Efficiency of the non-radiative energy transfer between tryptophan residues via the Förster mechanism [38] was evaluated as:

(4) $$E=\frac{1}{1+\frac{2/3}{\kappa^{2}}{\left(\frac{R}{R_{0}}\right)}^{6}}$$

where *R*_0_ is the Förster distance, *R* is the distance between the geometric centers of the indole rings of the donor and the acceptor, and κ^2^ is the factor of mutual orientation of the donor and the acceptor.

Orientation factor was determined in accordance with:

(5) $$\kappa^{2}={\left(\cos\theta -3\cos\theta_{A}\cos\theta_{D}\right)}^{2}$$

where θ is the angle between the directions of the emission oscillator of the donor and the absorption oscillator of the acceptor; θ_A_ and θ_D_ are the angles between the oscillators and the vector connecting the geometric centers of the donor and acceptor molecules [28].

The calculations were performed under the assumption of rigid oscillators. *R* and κ^2^ were calculated based on the atom coordinates. Due to the uncertainty of the donor quantum yield and the overlap integral value of the donor fluorescence and the acceptor absorption, *E* for the Trp-Trp energy transfer was calculated using *R*_0_ = 7.8 Å [26].

### 4.8. Molecular Dynamics of the Proteins

Molecular dynamics (MD) simulations were performed for the three-dimensional structures of the *V. harveyi* and *P. leiognathi* luciferases. The crystal structure of *V. harveyi* was downloaded from Protein Data Bank (PDB ID: 3FGC) in complex with FMN [13]. Initial coordinates of *P. leiognathi* luciferase were obtained by homology-based modeling using the SWISS-MODEL server and the crystal structure of *V. harveyi* luciferase as a template [39]. The sequence of *P. leiognathi* luciferases was retrieved from the UniProt database, including the *α*-subunit (P09140) and *β*-subunit (P09141) [40]. The high identity between the target and the template sequences (54.08 and 45.45% for *α* and *β*-subunits) ensured a high quality of the *P. leiognathi* luciferase three-dimensional model (GMQE = 0.78, QMEAN = ‒1.02) [9].

The missed disordered segment of the mobile loop of the *α*-subunit (283–290 amino acid residues) in the *V. harveyi* crystal was reconstructed by MODELLER [41]. Before starting the energy minimization step, FMN was removed from the 3FGC structure. MD simulations were conducted using GROMACS 5. 1. 4 with the CHARMM36 force field and the TIP3P explicit water. The following simulation protocol was the same for each protein [42,43,44]. The protein was centered in a cubic box with periodic boundary conditions at least 12 Å away from each of the box edges and solvated with water molecules. The net charge of the system was neutralized with sodium ions. Complete information about the system size is provided in Table S1.

The system was minimized with the steepest descent method (maximum force of 1000.0 kJ/mol). The cutoff distance for the short-range non-bonded interactions was 12 Å, and the electrostatic interactions were treated using the particle mesh Ewald method. An integration step of 2.0 fs was used, and bonds were constrained with the LINCS algorithm. The minimized system was heated and equilibrated at 300 K for 5 ns in the NVT ensemble with the restrained protein. The following NPT equilibrations were carried out in two stages. In the first phase, a 10 ns equilibration was performed with the restraint protein heavy atoms. In the second, a 10 ns equilibration followed, and all atoms in the system were free to move.

Production MD simulations were carried out in the NPT ensemble for 40 ns for each luciferase, collecting coordinates of the system every 10 ps. A modified Berendsen (V-rescale) thermostat and a Parrinello−Rahman barostat were employed.

Reconstruction of the movable loop segment in the 3FGC crystal, all visualizations and tryptophan alignment were performed using the UCSF CHIMERA software package [45]. The positions of amino acids in the luciferase structure is indicated in accordance with the sequence of *V. harveyi* luciferase.

The distance distributions between the mass centers of the two tryptophan indole rings were calculated using the gmx distance plugin in GROMACS. PONDR-VLXT was used to estimate the intrinsic disorders of both luciferase structures [33].

## 5. Conclusions

The present study has been carried out in the field of molecular biophysics and was directed at identifying factors affecting the conformational stability of proteins, as well as at developing methods of assessment and comparison of their structural stability. We have investigated the change of spectroscopic properties during urea-induced unfolding of two homologous proteins—*V. harveyi* and *P. leiognathi* luciferases—with special attention to the time-resolved fluorescence of these proteins. Despite the high percentage of identical and similar amino acids in their sequences, the studied enzymes belong to different subfamilies of bacterial luciferases with differing functionality, as previously shown by phylogenetic analysis. The results of our study indicate that the “fast” *P. leiognathi* luciferase has more stable tertiary and secondary structures than the “slow” *V. harveyi* luciferase. The most remarkable differences between the luciferases were revealed by time-resolved fluorescence in the range of 0–2 M of urea. We have analyzed the positions and the microenvironment of the tryptophan residues within the luciferase globule and found that in the *V. harveyi* protein they are packed more tightly. This enhances short-range interactions and aggravates quenching processes, leading to a decrease in the fluorescence lifetime. During the first stage of the urea-induced denaturation, both luciferases acquire the conformation of an inactive dimer, in which the quenching effects are removed and the difference between the proteins disappears. Our study has revealed that the tryptophans located in or nearby the C-terminal domain of the protein *α*-subunit (*α*W194, *α*W250, *α*W277) make the most significant contribution to the observed individual fluorescent properties of the bacterial luciferase. Meanwhile, the difference in the secondary structure stability could be due to the peculiarities of the sequence of the protein *β*-subunit. Previously, we demonstrated for carboanhydrase II that the two lifetimes of the protein fluorescence could probe different structural transitions, i.e., different stages of protein unfolding. In this work, we found for the bacterial luciferases that both fluorescence lifetimes report about the same structural transitions, but only using time-resolved parameters makes it possible to identify the difference in conformational stability of the two homologous proteins. However, to answer the question of whether the found properties of conformational stability is a sign of the entire subfamily (“fast” or “slow” luciferases) or is an individual feature of only the studied enzymes, further research is required.

## Figures and Tables

**Table 1:** Thermodynamic parameters of two stages of luciferase unfolding with urea. The parameters which differ for the two luciferases are in bold.

№	Method	Fraction of Transition ^#^		ΔG_H2O_, kcal/mol *		m_U_, kcal/M/mol *		[Urea]_50%_, M *	
		*P. l.*	*V. h.*	*P. l.*	*V. h.*	*P. l.*	*V. h.*	*P. l.*	*V. h.*
1	I_320_/I_360_	0.18 0.82	0.15 0.85	5.2 ± 2.3 **8.4** ± 0.7	1.6 ± 2.0 **6.6** ± 0.6	2.8 ± 1.3 2.0 ± 0.2	1.5 ± 0.3 1.7 ± 0.2	**1.8** ± 0.1 **4.2** ± 0.1	**1.0** ± 0.4 **3.9** ± 0.1
2	Θ_222_	0.27 0.73	0.34 0.66	4.2 ± 0.9 4.3 ± 0.6	4.1 ± 0.8 3.8 ± 0.6	3.2 ± 0.9 1.0 ± 0.2	3.0 ± 1.2 1.0 ± 0.3	1.3 ± 0.1 **4.4** ± 0.1	1.4 ± 0.2 **3.7** ± 0.2
3	τ_1_	0.30 0.70	0.39 0.61	**2.1** ± 0.3 **10.8** ± 0.9	**4.0** ± 0.6 **8.2** ± 0.7	**2.0** ± 0.3 2.4 ± 0.2	**4.4** ± 0.7 2.0 ± 0.2	1.0 ± 0.1 **4.5** ± 0.1	0.9 ± 0.1 **4.0** ± 0.1
4	τ_2_	0.70 0.30	0.73 0.27	2.5 ± 0.4 11.0 ± 4.3	3.5 ± 0.8 8.5 ± 4.2	**2.3** ± 0.3 2.4 ± 0.9	**3.9** ± 0.9 2.0 ± 1.0	1.1 ± 0.1 **4.6** ± 0.2	0.9 ± 0.1 **4.2** ± 0.2

^#^ Calculated as the ratio of parameter change in the individual transition to the total parameter change upon denaturation. * Calculated from the fitting curves in Figure 1A and Figure 2.

**Table 2:** Microenvironment characteristics of tryptophan residues in *V. harveyi* (*V.h.*) and *P. leiognathi* (*P.l.*) luciferase structures in their native state. The most remarkable differences between the proteins are in bold.

Tryptophan ↓		Characteristics of Microenvironment in 7 Å * ^&^										SASA, Å^2^ (%) ^#^	
		N_Σ_, pcs		N_pol_, pcs, (%) ^&^		N_sec_ (N_α_ + N_β_), pcs, (%) ^&^		N_C-term_, pcs, (%) ^&^		N_β-sub_, pcs, (%) ^&^			
*Subunit*	*Position*	*P.l.*	*V.h.*	*P.l.*	*V.h.*	*P.l.*	*V.h.*	*P.l.*	*V.h.*	*P.l.*	*V.h.*	*P.l.*	*V.h.*
*α*	40	159	177	64 (40)	63 (36)	159 (5 + 154), (100)	170 (0 + 170), (96)	14 (9)	18 (10)	0	0	14.8 ± 7.9 (5.6 ± 3.0)	8.6 ± 6.2 (3.2 ± 2.4)
*α*	131	-	162	-	59 (36)	-	99 (84 + 15), (61)	-	12 (7)	-	0	-	7.2 ± 5.9 (2.7 ± 2.2)
*α*	182	135	148	54 (40)	56 (38)	121 (107 + 14), (90)	130 (111 + 19), (88)	0	0	0	0	25.4 ± 13.1 (9.6 ± 5.0)	33.0 ± 8.3 (12.5 ± 3.2)
*α*	194	127	141	39 (31)	44 (31)	78 (34 + 44), (61)	84 (36 + 48), (60)	50 (39)	60 (43)	0	0	19.3 ± 7.9 (7.3 ± 3.0)	20.8 ± 7.9 (7.9 ± 3.0)
*α*	250	**121**	**151**	33 (27)	48 (32)	79 (79 + 0), (65)	83 (82 + 1), (55)	93 (77)	106 (70)	0	0	39.8 ± 11.7 (15.1 ± 4.4)	34.3 ± 13.6 (13.0 ± 5.1)
*α*	277	**93**	**121**	39 (42)	52 (43)	**67** (0 + 67), (72)	**20** (20 + 0), (17)	63 (68)	64 (53)	**28** (30)	**0**	114.2 ± 22.8 (43.3 ± 8.6)	99.4 ± 26.8 (37.7 ± 10.2)
*β*	182	146	148	48 (32)	47 (32)	118 (101 + 17), (81)	132 (111 + 21), (89)	0	0	N/A	N/A	25.7 ± 10.3 (9.7 ± 3.9)	22.6 ± 9.1 (8.6 ± 3.5)
*β*	194	145	152	41 (28)	44 (29)	87 (42 + 45), (60)	92 (38 + 54), (61)	0	0	N/A	N/A	17.8 ± 6.5 (6.7 ± 2.5)	6.1 ± 5.1 (2.3 ± 1.9)

* N_Σ_—total number of protein atoms; N_pol_—number of polar atoms; N_sec_ (N_α_ + N_β_)—number of the atoms included in secondary structure elements (α-helix + β strands); N_C-term_—number of atoms of the C-terminal domain of the protein *α*-subunit; N_β-sub_—number of atoms of the protein *β*-subunit; SASA—solvent-accessible surface area (average value during MD simulation ± standard deviation); ^&^ in parentheses, the percentage of the corresponding atoms from the total number of atoms (N_Σ_) is shown; ^#^ in parentheses, the percentage of the solvent-accessible surface area for the analyzed tryptophan residue from the maximal SASA for tryptophan in a protein is shown.

## Data Availability

The data presented in this study are available on request from the corresponding author.

## Supplementary Materials

The following are available online at https://www.mdpi.com/article/10.3390/ijms221910449/s1.
