# Supplementary material for: Bacterial Luciferases from Vibrio harveyi and Photobacterium leiognathi Demonstrate Different Conformational Stability as Detected by Time-Resolved Fluorescence Spectroscopy
Source: Int J Mol Sci. 2021 Sep 28;22(19):10449. doi: 10.3390/ijms221910449 (PMC8508739; doi:10.3390/ijms221910449)
Supplement: Supplementary file 1 [file ijms-22-10449-s001.zip › ijms-1381484-supplementary.pdf]

Bacterial luciferases from *Vibrio harveyi* and *Photobacterium leiognathi* demonstrate different conformational stability as detected by time-resolved fluorescence spectroscopy

Elena V. Nemtseva [1,2,*], Dmitry V. Gulnov [1], Marina A. Gerasimova [1], Lev A. Sukovatyi [1], Ludmila P. Burakova [1,2], Natalia E. Karuzina [1], Bogdan S. Melnik [3], Valentina A. Kratasyuk [1,2]

[1] Siberian Federal University, 660041, Svobodny 79, Krasnoyarsk, Russia

[2] Institute of Biophysics SB RAS, 660036, Akademgorodok 50/50, Krasnoyarsk, Russia

[3] Institute of Protein Research, Russian Academy of Sciences, 142290, Institutskaya 4, Pushchino, Moscow Region, Russia

Supplementary Materials

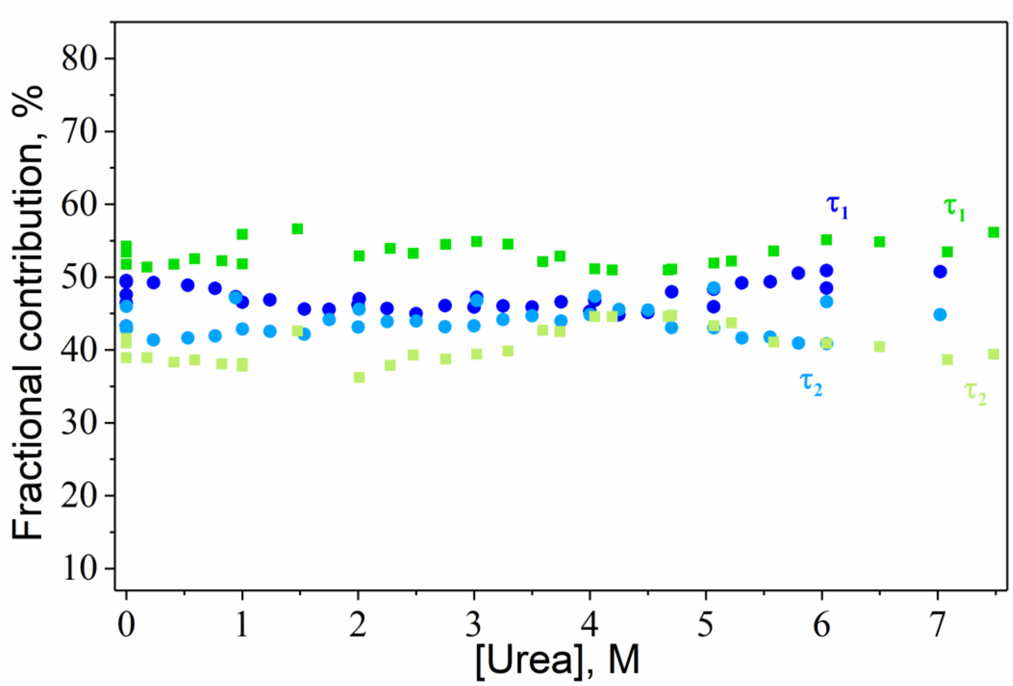

Figure S1. The fractional contribution of the lifetime components (DAS) in fluorescence spectra of the *V. harveyi* (green) and the *P. leiognathi* (blue) luciferases at various urea concentrations

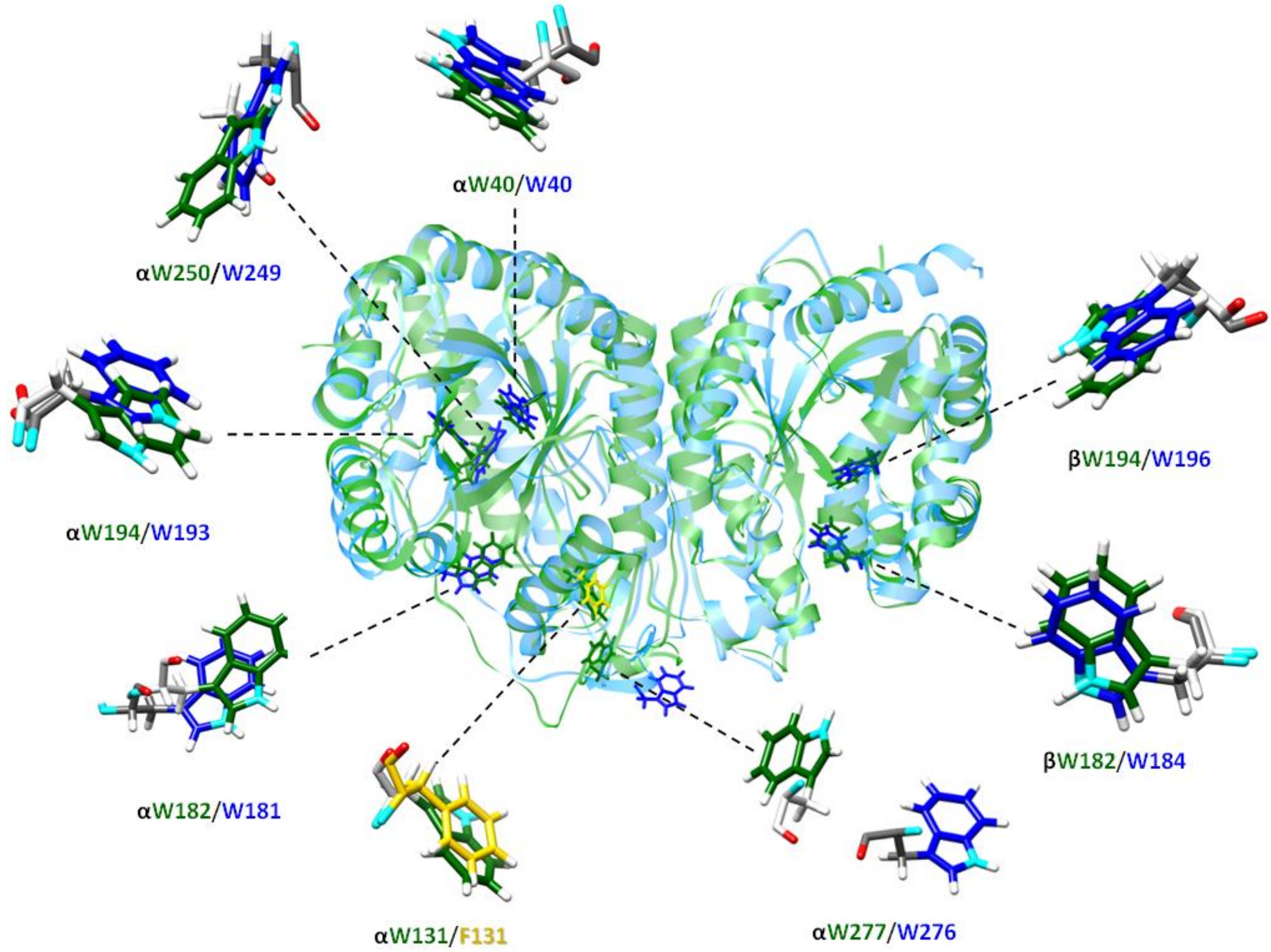

Figure S2. The relative positions of tryptophans of *V. harveyi* (green) and *P. leiognathi* (blue) luciferases after tertiary structure alignment. In the position 131 of *P. leiognathi* α-subunit there is phenylalanine (yellow). Tryptophan position is indicated basing on the individual sequence of the protein.

Details of molecular dynamics simulation

Table S1: The parameters of the systems used for MD simulations of the *Vibrio harveyi* (*V.h.*) and the *Photobacterium leiognathi* (*P.l.*) luciferases

Protein	Number of water molecules. pcs	Number of Na^+ ions, pcs	Total number of atoms in the system, pcs	Equilibrated cell size, Å
P. l.	42505	30	138296	110.7 × 110.7 × 110.7
V. h.	40821	36	132922	109.2 ×109.2 × 109.2
